# The effect of unsteady streamflow and stream-groundwater interactions on oxygen consumption in a sandy streambed

**DOI:** 10.1038/s41598-019-56289-y

**Published:** 2019-12-24

**Authors:** Jason Galloway, Aryeh Fox, Jörg Lewandowski, Shai Arnon

**Affiliations:** 1 Leibniz-Institute of Freshwater Ecology and Inland Fisheries, Department Ecohydrology, Berlin, D-12587 Germany; 2 Humboldt University Berlin, Geography Department, Berlin, D-12489 Germany; 3 Ben-Gurion University of the Negev, The Jacob Blaustein Institute for Desert Research, Zuckerberg Institute for Water Research, Sde-Boker, 84990 Israel

**Keywords:** Element cycles, Pollution remediation

## Abstract

Streamflow dynamics are often ignored when studying biogeochemical processes in the hyporheic zone. We explored the interactive effects of unsteady streamflow and groundwater fluxes on the delivery and consumption of oxygen within the hyporheic zone using a recirculating flume packed with natural sandy sediments. The flume was equipped with a programmable streamflow control and drainage system that was used to impose losing and gaining fluxes. Tracer tests were used to measure hyporheic exchange flux and a planar optode was used to measure subsurface oxygen concentration patterns. It was found that the volume of the oxic zone decreased when the losing flux declined, and was drastically decreased when gaining conditions were applied. It was also found that unsteady streamflow led to a slight increase in the average volume of the oxic zone, compared to the average volume of the oxic zone under steady streamflow. However, the average oxygen consumption rates were significantly higher under unsteady streamflow compared to steady streamflow under all groundwater conditions with the exception of the highest losing flux. The present study provides the first insight into the interactions between streamflow unsteadiness and losing/gaining fluxes and improve understanding of their impact on microbial metabolism in the hyporheic zone.

## Introduction

Aerobic respiration has a strong impact on the overall biogeochemistry of river sediments, and ultimately on the water quality and ecology of freshwater systems^1^. Aerobic respiration in sandy streambeds is often concentrated in the upper section of the sediment (benthic zone) and in the hyporheic zone (HZ), which is the area directly beneath and adjacent to a river where streamwater enters saturated sediments for a period of time before returning to the river^2^. The HZ is of increasing research interest due to its high potential for nutrient removal^3,4^ and attenuation of pollutants^2,5,6^. The HZ is considered to be a dynamic ecotone that provides various ecosystems services^7–9^. Hyporheic flow can be induced by different mechanisms and geomorphic structures including pool-riffle sequences, ripples, meander bends, obstacles such as dead wood and obstacles in the streambed^6^. While all types of hyporheic exchange fluxes play an important role in the attenuation and tranformation of pollutants, bedform-driven hyporheic exchange plays a particularly prominent role due to the relatively short flow paths and higher microbial activity compared to other types of hyporheic exchange^4^. The quantity of stream water per unit of time which flows into the streambed and returns to the stream after a certain period of time in the subsurface is termed hyporheic exchange flux (HEF). HEF has been extensively studied in the last decade and the hydromechanical processes which drive HEF are well-understood^10^. In general, riverbed morphology, streambed hydraulic characteristics, streamflow and stream-groundwater interactions have been highlighted as key drivers of bedform-scale HEF^11–13^. Therefore any perturbations to streamflow can be expected to impact HEF and biogeochemical processes which are dependent on the delivery of advectively transported solutes such as oxygen.

Experimental and modelling studies on solute dynamics in HZs have been traditionally conducted using steady streamflow^14,15^. However, streamflow regimes in natural systems are rarely steady. Many natural systems are subject to large, and often, systematic fluctuations in streamflow driven by processes including: diel variations in snow melt^16^, the impacts of dams and hydropower plants^17^, tides^18^, floods^19^ and discharge cycles from wastewater treatment plants (See Supplementary Fig. S1). Modelling studies into the possible impacts of neglecting streamflow unsteadiness when studying HZ processes have suggested the potential for systematic underestimation of HEF^19,20^. However, despite the ubiquitous cases of streamflow dynamics, only recently have research begun to explore the effect of streamflow fluctuations on nutrient dynamics.

For example, Kaufman *et al*.^21^ demonstrated the importance of streamflow perturbations on subsurface oxygen dynamics in series of flume experiments using step changes in streamflow and suggested that streamflow perturbations have important impacts on microbial metabolism. The change in streamflow conditions is usually associated with a change in water level, which ultimately affects the hydraulic gradient between the stream and the groundwater, and the associated net water exchange in the form of losing or gaining fluxes (describing the cases when the net water flow direction is leaving or entering the stream, respectively).

Recent experimental and modelling advances have allowed the study of stream-groundwater interactions in more detail. It was found that increasing the magnitude of losing or gaining fluxes reduced HEF in an exponential manner and this decline was similar for a specific flux regardless of its vertical direction (i.e., similar for both losing and gaining fluxes of the same magnitude)^22^. In addition, De Falco *et al*.^23^ and Trauth *et al*.^24^ revealed that increasing losing and gaining fluxes under steady streamflow resulted in nonlinear changes in oxygen consumption rates (OCRs) with OCRs being either enhanced or reduce depending on the specific set of conditions present. It is widely accepted today that HEF is determined by the competitive interaction between streamflow and groundwater losing and gaining conditions, however to the best of our knowledge the impacts of unsteady streamflow and groundwater interactions on microbially mediated prsocess has never been studied.

The main objective of the present study was to quantify the effects of unsteady streamflow (${V}_{unsteady}$) on OCRs under a range of losing and gaining fluxes (q${}_{gw}$). The mean of the ${V}_{unsteady}$ sinusoid was used as steady streamflow (${V}_{steady}$) for comparison.

We hypothesized that relative to steady streamflow, unsteady streamflow will lead to: 1) a net increase in the volume of the oxic zone (${{\rm{V}}}_{{\rm{oxic}}}$) and 2) an increase in OCRs. We expect that during unsteady streamflow a net increase in the flux of dissolved oxygen to the streambed will be observed due to the nonlinear relationship between streamflow and HEF leading to periods of increased streamflow causing a greater increase in HEF than the corresponding decrease in HEF under periods of decreased streamflow. We postulated that the variable redox conditions under unsteady streamflow will have a stimulatory effect on microbial communities leading to higher OCRs. Finally, we also hypothesized that the larger the magnitude of the losing or gaining flux (${{\rm{q}}}_{{\rm{gw}}}$), the more similar V${}_{{\rm{oxic}}}$ and OCRs will be between tests conducted under steady and unsteady streamflow as the dominant driver of HEF in the system shifts from being streamflow to q${}_{{\rm{gw}}}$.

## Results

### HEF under different streamflow and losing fluxes

**Figure 1:**
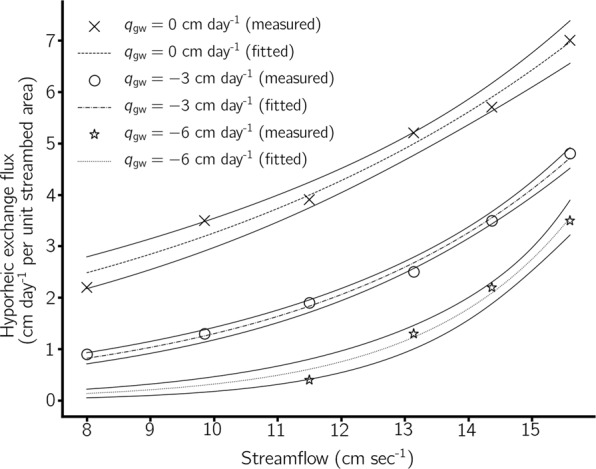
Hyporheic exchange flux increases exponentially with increasing streamflow, and diminishes with increasing magnitude of losing flux. Dashed lines indicate the exponential fit and solid lines indicate the 95% confidence intervals. Tests were only conducted under gaining conditions as previous theoretical and experimental research has conclusively shown that HEF is the same regardless whether a losing or gaining flux is applied^22^. This might be counter-intuitive but keep in mind that according to the definition given in the introduction subsurface flow is only classified as HEF if flow paths begin and end at the sediment-water interface. Fitted parameter values for each line are provided in Supplementary Information Table S2.

HEF increased substantially with increasing streamflow under neutral and losing conditions (Fig. 1). HEF under gaining conditions was not tested as previous research has shown HEF under losing and gaining conditions to be the same for a given vertical flux^22^. In addition, increasing the magnitude of the losing flux reduced HEF relative to neutral conditions (q${}_{{\rm{gw}}}$ = 0 cm day${}^{-1}$). The relationship between HEF and streamflow became increasingly exponential (i.e. the size of exponent, *b* in Eq. 1, increased) as the losing flux was increased.

### The effect of flow conditions on V${}_{{\bf{oxic}}}$

**Figure 2:**
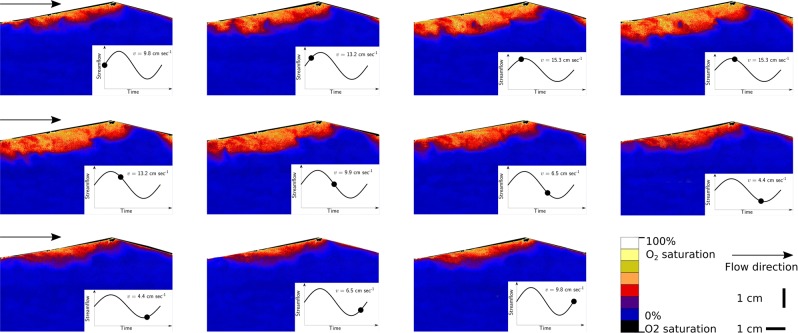
Optode image sequence showing the spatial and temporal change in subsurface oxygen concentration (% saturation) when streamflow was varied using a sinusoidal function with a period of 10 hours (see figure insets) with no imposed groundwater flux. *v* denotes streamflow.

**Figure 3:**
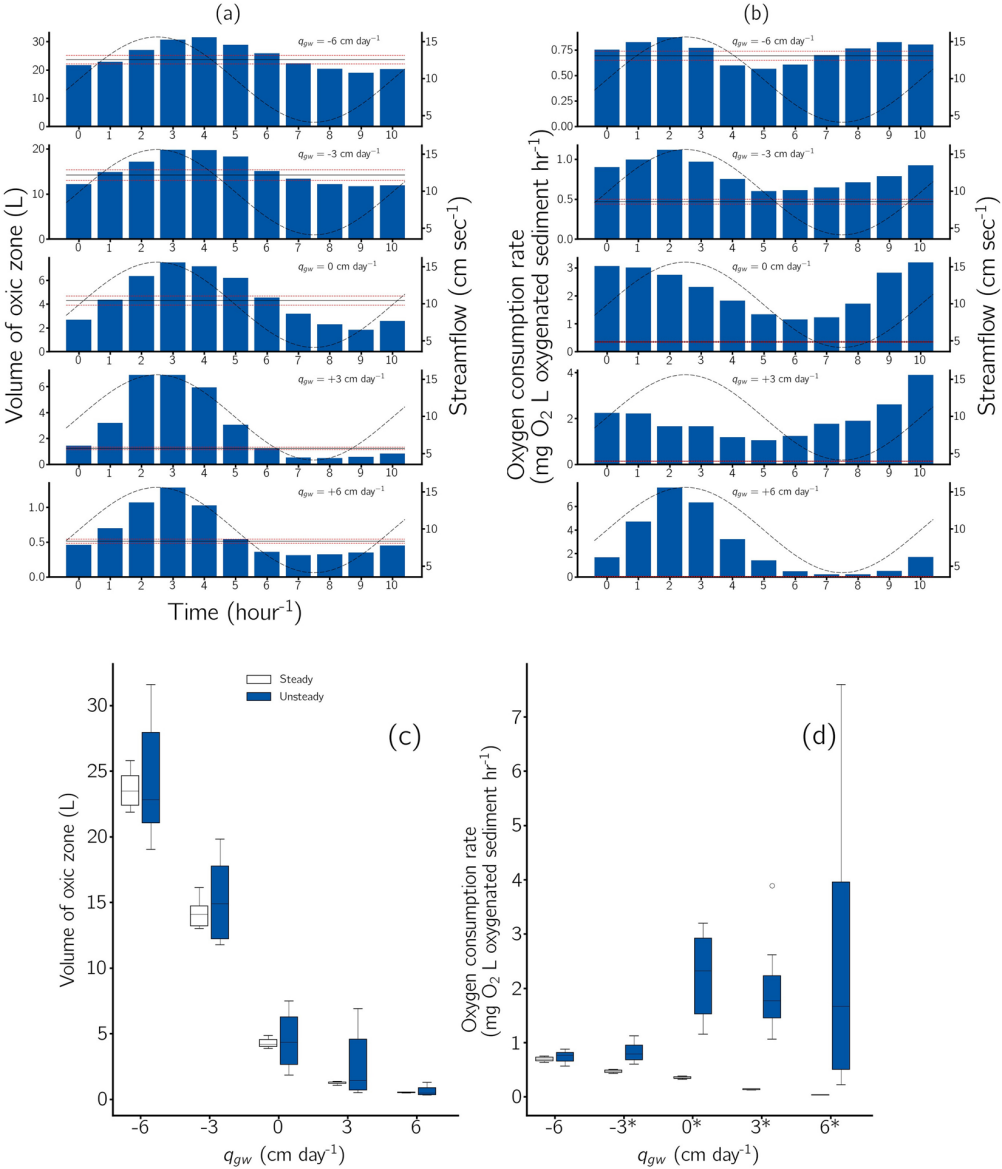
Volume of the oxic zone (**a**) and oxygen consumption rates (**b**) during 10-hour test period during steady and unsteady streamflow in the presence of various losing and gaining fluxes. Measurements were taken hourly. Bars denote the volume of the oxic zone (**a**) or oxygen consumption rate (**b**) under unsteady streamflow. Solid lines denote the mean volume of the oxic zone (**a**) and the mean oxygen consumption rate (**b**) under steady streamflow. The dashed sinusoidal lines denote streamflow and red lines show standard deviations under steady streamflow. Note that the scale on the y-axis varies between figures. Box-and-whisker plot showing the total volume of the oxic zone (**c**) and oxygen consumption rates (**d**) during each 10-hour period during steady (white, $n=11$) and unsteady conditions (blue, $n=6$). Variability within each test is indicated by the following: the central line shows the median, the upper and lower boundaries of the box indicate the 25${}^{{\rm{th}}}$ and 75${}^{{\rm{th}}}$ percentile values, the error bars indicate the 10${}^{{\rm{th}}}$ and 90${}^{{\rm{th}}}$ percentile values and the circle represents outliers. The * denotes a statistically significant difference in median values within steady and unsteady pairs (*p*-value = 0.05).

Oxygen concentration measurements showed that V${}_{{\rm{oxic}}}$ (defined here as the volume of subsurface sediment with an oxygen saturation $>$ 15%, upscaled from a single sand dune 2D image to the entire length and width of the flume) was relatively constant under steady streamflow with no groundwater interaction (q${}_{{\rm{gw}}}$ = 0 cm day${}^{-1}$) but varied during unsteady streamflow (Table 1, Figs. 2 and 3(a,c)). In the presence of groundwater interaction this trend was still present, regardless of the direction of the flux applied, i.e. greater variation in V${}_{{\rm{oxic}}}$ was always observed during unsteady streamflow than during steady streamflow at a given losing or gaining flux (Table 1). As the imposed groundwater flux went from the strongest losing condition to the strongest gaining condition generally the variation in mean V${}_{{\rm{oxic}}}$ decreased for the same streamflow condition. This is reflected by the decreasing values of the standard deviation with the exception of the unsteady flow test at a gaining flux +3 cm day ${}^{-1}$ (Table 1). However, results of Welch two sample *T*-tests showed no statistically significant differences between V${}_{{\rm{oxic}}}$ under steady and unsteady streamflow at any of the losing or gaining fluxes tested (Table 1).

**Table 1:** Welch Two Sample *T*-test results for steady streamflow (n = 6) and unsteady streamflow (n = 11) tests, with the associated *p*-values, the mean time-integrated volumes of the oxic zone, oxygen consumption rates and standard deviations (S.D.). Each test had a duration of 10 hours. d.f. denotes degrees of freedom, * denotes a statistically significant result (*p*-value < 0.05). ${}^{\dagger }$One outlier was removed before subsequent calculations.

q${}_{{\rm{gw}}}$	*T*-statistic	*p*-value	d.f.	Mean steady	Steady S.D.	Mean unsteady	Unsteady S.D.
	**Volume of oxic zone (L)**						
$-6$	$-0.67$	0.51	13.61	23.61	1.55	24.61	4.43
$-3$	$-0.90$	0.39	13.99	14.20	1.19	15.15	3.15
0	$-0.22$	0.83	11.21	4.29	0.38	4.43	2.07
+3	$-2.03$	0.07	10.06	1.23	0.10	2.82	2.60
+6	$-1.08$	0.30	10.31	0.51	0.03	0.63	0.35
	**Oxygen consumption rate** (mg ${{\rm{O}}}_{2}$ L hr${}^{-1}$)						
$-6$	$-1.16$	0.27	14.58	0.69	0.04	0.74	0.10
$$-3* $$	$$-6.58$$	$$ < $$0.01	11.09	0.47	0.03	0.82	0.17
0*	$-7.84$	$<$0.01	10.03	0.35	0.02	2.22	0.79
+3*$\dagger$	$-6.70$	$<$0.01	9.00	0.14	0.01	1.92	0.84
+6*	$-3.23$	$<$0.01	10.00	0.03	0.00	2.56	2.59

V${}_{{\rm{oxic}}}$ under steady streamflow without groundwater interaction (q${}_{{\rm{gw}}}$ = 0 cm day${}^{-1}$) varied slightly around the time-integrated median (Table. 1, Fig. 3(a,c)). This variability was generally reduced from the strongest losing flux (q${}_{{\rm{gw}}}$ = $-6$ cm day${}^{-1}$) to the strongest gaining flux (q${}_{{\rm{gw}}}$ = +6) (Table. 1, Fig. 3(a,c)). In addition, at the strongest losing flux (q${}_{{\rm{gw}}}$ of $-6$ cm day${}^{-1}$), the delay (or phase shift) in V${}_{{\rm{oxic}}}$ with reference to the flow sinousoid was 50 min${}^{-1}$ while at the strongest gaining flux the delay was 18 mins${}^{-1}$ (Supplementary Information Fig. S2). The sinusoidal unsteady flow varied between 4.38 cm sec ${}^{-1}$ and 15.3 cm sec${}^{-1}$ with an average flow of 9.9 cm sec${}^{-1}$. Under neutral conditions (q${}_{{\rm{IF}}}$ = 0 cm day${}^{-1}$) the volume of the oxic zone varied also in a sinusoidal pattern between 1.9 L and 7.9 L with an average of 2.7 L. The sinusoidal function of the volume of the oxic zone followed the sinusoidal function of unsteady flow with some delay (Fig. 3, Supplementary Information Fig. S2).

### The effect of flow conditions on OCR

The variability of OCRs during unsteady streamflow was much larger than during steady streamflow conditions (Fig. 3(d)). The range of variation in OCRs under unsteady streamflow increased from 0.57 - 0.88 mg $${{\rm{O}}}_{2}$$ $${{\rm{hr}}}^{-1}$$ at intense losing conditions ($${{\rm{q}}}_{{\rm{gw}}}=-6$$ cm day$${}^{-1}$$) to 0.22 - 7.60 mg $${{\rm{O}}}_{2}$$$${{\rm{hr}}}^{-1}$$ at intense gaining conditions ($${{\rm{q}}}_{{\rm{gw}}}=+6$$ cm day$${}^{-1}$$)(Fig. 3(b,d)). In general higher OCRs were associated with higher overlying water velocities and under stronger gaining conditions, however, the temporal trends in OCRs were less closely associated with streamflow than $${{\rm{V}}}_{{\rm{oxic}}}$$ (Fig. 3(b)). At the largest losing flux ($${{\rm{q}}}_{{\rm{gw}}}=-6$$ cm day$${}_{-1}$$) mean OCRs of steady and unsteady streamflow were not significantly different (*t*-statistic = $$-1.16$$, *p*-value = 0.27). However for all other losing and gaining fluxes, unsteady streamflow led to significantly higher OCRs (*p*-value = < 0.05) (Table 1).

## Discussion

### Response of V${}_{{\rm{oxic}}}$ to acceleration and deceleration

**Figure 4:**
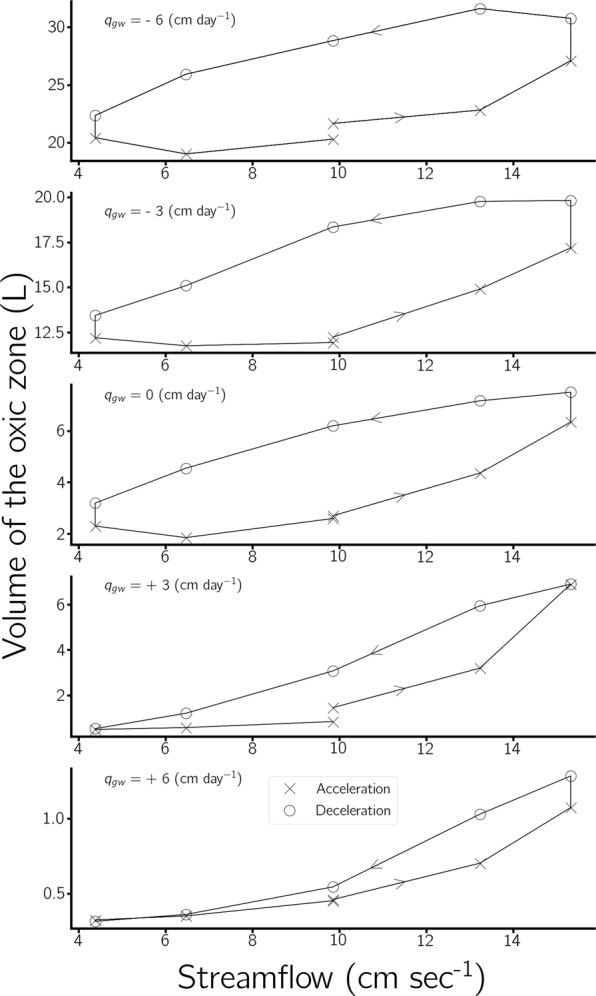
Volume of the oxic zone at different streamflows for runs with different imposed groundwater fluxes. The volume of the oxic zone is dependent upon antecedent streamflow. Markers denote antecedent acceleration ($n=6$) and deceleration ($n=5$). The volume of the oxic zone is generally larger at the same streamflow during deceleration.

V${}_{{\rm{oxic}}}$ increased and decreased following streamflow changes, but with some delay (Figs. 2 and 3(a)). Figure 4 visualizes that streamflow determines V${}_{{\rm{oxic}}}$. However, V${}_{{\rm{oxic}}}$ does not only depend on the current streamflow but also on the previous streamflow. The delay in the V${}_{{\rm{oxic}}}$ shrinkage under deceleration of streamflow is consistently longer than the delay of V${}_{{\rm{oxic}}}$ expansion after acceleration of streamflow (Figs. 2 and 4). This behaviour is in accordance with findings of Kaufman *et al*.^21^, which were obtained in flume experiments using a simple step function to modulate between two distinct streamflows, which represented low and high streamflow conditions. This highlights that the impacts of streamflow acceleration and deceleration on the HZ are not simply mirror-inverted situations (Fig. 4). Instead, there are complex physical, chemical and biological process interactions. Increases in V${}_{{\rm{oxic}}}$ following acceleration are driven by increases in the pressure gradient between the surface and subsurface which immediately increases advective pumping. Thus the dominant time-determining process is the physical transport of oxygen into deeper horizons of the HZ. The implication of this nonlinearity is that the change in advective transport under changing velocities will also be nonlinear^11^ (Fig. 1). After deceleration of streamflow there is less transport of oxygen into deep sediment layers, however, the decreased transport of oxygen into the HZ does not result immediately in a smaller V${}_{{\rm{oxic}}}$ because the main processes which reduce oxygen in the subsurface are biogeochemical processes such as aerobic respiration. In other words, the time needed for microbial communities to consume the oxygen present in the pore water causes the increased delay in the response of the subsurface to decreases of streamflow, while under streamflow acceleration it is the combination of microbial activity and oxygen transport. Given that HEF increases proportionally to streamflow in an exponential manner (Eq.1 and Table S2), one unit increase in streamflow causes a larger increase in HEF than the corresponding one unit decrease in streamflow^22^.

### The impact of stream-groundwater interactions in the presence of unsteady streamflow on V${}_{{\bf{oxic}}}$

The fluxes to and from groundwater can enhance or suppress the effect of unsteady streamflow on V${}_{{\rm{oxic}}}$. Relative to tests where no stream-groundwater interaction was present (q${}_{{\rm{gw}}}$ = 0 cm day${}^{-1}$), the imposition of a vertical flux towards groundwater (i.e. losing conditions) caused V${}_{{\rm{oxic}}}$ to follow changes in streamflow with a longer delay (Figs. 3(a) and S2), whilst the inverse was true when an upward vertical flux was imposed (i.e. gaining conditions). The explanation for this is that when no groundwater fluxes are present, HEF is driven by the pressure gradient caused by water flowing over bed morphology^22,25^ thus any variations to streamflow result in a corresponding increase or decrease in HEF. When a losing flux is imposed on the system, advective flow penetrates deeper into the streambed compared to only bedform-induced HEF. Thus the total flux into the streambed depends not only on hyporheic flow, but also on the losing flux the effect of which are delayed due to the longer distances that oxygen needs to be transported into the streambed. Under gaining conditions the exchange is driven solely by bedform-induced HEF. However, because the hyporheic zone size is reduced due to the upward flow, the transport distances are small^22,24^, i.e. the response between streamflow and V${}_{{\rm{oxic}}}$ is tightly coupled such that the maximum and minimum of both occur almost simultaneously (Supplementary Information Fig. S2).

In summary, the often used simplification of using steady mean streamflow in modelling^26,27^ and flume studies^15^ instead of a dynamic regime will systematically underestimate the amount of advectively transported solutes delivered to hyporheic zones. However the severity of this underestimation will depend on the amount of unsteadiness in streamflow and the magnitude of vertical fluxes present in addition to other physical characteristics such as the morphology of the streambed and its roughness. Under losing conditions, streamflow changes can lead to relatively minor changes in V${}_{{\rm{oxic}}}$ compared to gaining conditions. Whereas, V${}_{{\rm{oxic}}}$ is highly sensitive to the vertical flux and can be drastically reduced with only a slight shift from losing to gaining flow conditions.

### The impact of stream-groundwater interactions in the presence of unsteady streamflow on OCRs

During steady streamflow not only is V${}_{{\rm{oxic}}}$ relatively constant but also its location in space remains similar. Thus, when considering a discrete location in the sediment it is either permanently oxic or permanently anoxic. During unsteady streamflow there are zones in the HZ that are also either permanently oxic or permanently anoxic (Fig. 2), while other locations are subject to transient oxic and anoxic conditions. In the presence of unsteady streamflow we observed significantly higher OCRs for all tests apart from the strongest stream losing condition (q${}_{{\rm{gw}}}$ = $-6$ cm day${}^{-1}$) compared to steady streamflow (Fig. 3(c) and Table 1). The observed increases in OCRs can again be explained by the nonlinear relationship between HEF and streamflow, similar to V${}_{{\rm{oxic}}}$. Nonlinear increases in HEF delivers more oxygen to the streambed, which is consumed by the microoganisms present. Non-statistically significant differences ($p > 0.05$) in OCRs between steady and unsteady streamflow when a losing flux of 6 cm day${}^{-1}$ was imposed were probably due to the fact that the system was dominated by the losing flux and thus unsteady streamflow was of secondary importance^22^.

When the vertical flux changes from losing to neutral and further to gaining, OCRs under unsteady streamflow becomes higher compared to the corresponding OCRs under steady streamflow (Fig. 3(d) and Table 1). However it should be noted that due to the reduced volume of the oxic zone, the total respiration is less under gaining conditions than under losing conditions. In addition, when moving from losing towards gaining conditions, the vertical flux increases the relative importance of streamflow and thus OCRs show higher variability around the average. This is likely caused a by several factors. First, the uppermost layer of the streambed has a larger biomass and more active biofilms than deeper layers^28^ and where q${}_{{\rm{gw}}}$ was $\ge$ 0 cm day${}^{-1}$ this volume represents a larger proportion of the volume of the sediment where HEF occurs. Second, as streamflow is the main driver for HEF, unsteady streamflow produces proportionally greater variation in oxic/anoxic conditions compared to losing conditions when the total volume of the oxic zone is considered as a whole. Finally, alternating oxic/anoxic conditions have been shown to promote increased microbial diversity^29^ and have been suggested to lead to enhanced rates of microbially-mediated redox reactions^30,31^. In addition, transient oxic/anoxic conditions increase the potential of streams biotransformative capacity as the removal of many micropollutants can only occur under oxic or anoxic conditions^2,32^. Thus improving understanding of the impacts of unsteady streamflow will be important for stream restoration in wastewater-impacted streams that are subject to high loads of micropollutants^5,33^.

### Field implications

Flow and biogeochemical dynamics are coupled, and increasing numbers of studies have explored this relationship in the last decade due to the increasing availability of continuous data from reliable and relatively inexpensive sensors for monitoring water quality parameters^34^. Continuous data of stream discharge and nutrients such as oxygen, DOC, and nitrate are the focus of those studies^35–37^. Analysis has focused on discharge-concentration relationships searching for trends, empirical connection and hysteresis, and various conceptual models have been developed in order to explain the dominant processes that control biogeochemical process dynamics in streams^36,38^. On one hand, none of these models has explicitly incorporated the hyporheic zone and exchange processes, nor the interaction between HEF and losing or gaining fluxes. On the other hand, other models suggest that the hyporheic zone is the most active zone within stream networks, and can be used to predict, for example, denitrification^4^. The observations from our experiments suggest that the effect of groundwater could potentially be much more than currently anticipated by just including dilution or nutrient input. Such interactions may help in deciphering current mismatches of discharge-concentration relationships and thus we postulate that tighter coupling between different modelling approaches are needed to fully capture the complex process interaction that affect stream metabolism. Finally it should be noted that the increased OCRs observed during stream gaining conditions in our experiment under laboratory conditions could potentially be enhanced or diminished in field conditions. Respiration rates are temperature-dependent and thus the temperature of upwelling groundwater relative to stream water could increase or decrease respiration rates depending on the season^39^. In general during summer months groundwater is cooler relative to stream water and upwelling groundwater would be expected to reduce OCRs while the inverse is true during winter months. The extent to which the temperature of upwelling groundwater will enhance or reduce respiration rates is likely to be dependent on specific site conditions. For example, the relative difference in temperature between stream and groundwater, the strength of the vertical flux and sediment properties such as thermal conductivity and porosity of the sediments through which the water is flowing. Our results show that the interactions with groundwater could potentially be critical for oxygen respiration, especially under gaining conditions. While in most of the cases oxygen was fully consumed, and thus, the relationship between oxygen consumption rates and HEF could be probably be derived with good predictive accuracy. However, respiration zones differed significantly in location and size, while low oxygen zones differed drastically between cases (Fig. 2). The spatial distribution of oxic/anoxic zones has the potential to influence processes that are activated by anaerobic conditions. For example, denitrification^40^, metal mobility^41^, and redox-related transformation of organic contaminants^2,42^. In addition, redox zonation is expected to influence benthic dwelling organisms and the sedimentary ecosystem as a whole^43,44^. While these processes were beyond the scope of the present work, it important to note that since any field sampling within the streambed should take into account the observed hot spots and hot moments that are strongly affected by the interactions between the stream and the groundwater.

## Conclusions

This work has shown that the common practice of using steady streamflow as a basis for investigations of biogeochemical processes in natural systems can lead to a systematic misrepresentation of redox sensitive processes. For the first time, we demonstrate the complex competitive interaction between unsteady streamflow, groundwater and their impact on oxygen consumption. Our findings reveal that V${}_{{\rm{oxic}}}$ and OCRs are increasingly sensitive to streamflow perturbations as losing fluxes diminish and gaining fluxes increase. We also show that the timing and locations of the active zones within stream sediments are not the same under losing and gaining conditions. Finally, we demonstrate that the V${}_{{\rm{oxic}}}$ drastically reduces as conditions shift from losing to gaining flow conditions, but OCR behaves differently. Initially, when losing conditions are diminished, OCR changes only slightly. However, a further shift into gaining conditions decreases V${}_{{\rm{oxic}}}$ but increases OCR levels and variability. These patterns are intrinsically linked to the nonlinear relationship between streamflow conditions and HEF and are expected to be consistent in streambeds with high microbial activity. Further research should focus on examining the impact of how the nature of streamflow unsteadiness such as periodicity and amplitude of fluctuations can impact the delivery of advectively transported solutes to the subsurface and the processing of redox-sensitive biogeochemical processes and additionally how these relationships are affected by ambient groundwater temperature. The results of the present study will enable better informed decisions to be taken when designing studies which aim to examine biogeochemical processes within the HZ.

## Methods

### Experimental system

**Figure 5:**
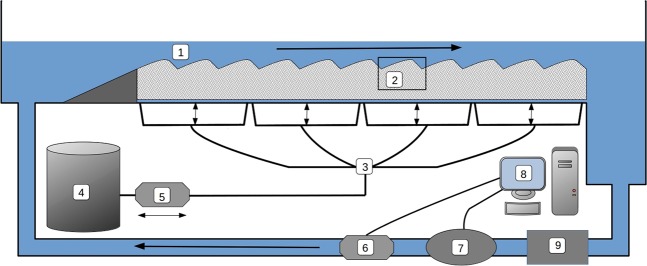
Schematic diagram of the flume. The numbers show the main channel (1), the optode film attached to flume wall (2), the vertical flux system (3), a storage/drainage tank of the vertical flux system (4), the peristaltic pump used to impose losing/gaining fluxes (5), the centrifugal pump connected to a computer used to control streamflow (6), a flow meter (7), the PC used to control pump speed based on flow meter readings (8) and the chiller used to maintain a constant water temperature (9).

**Figure 6:**
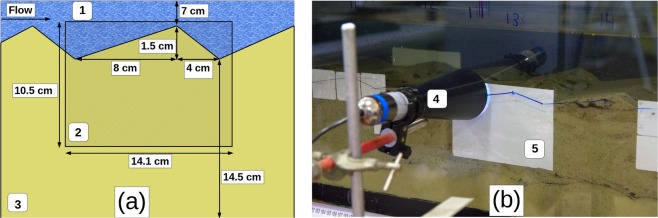
Schematic representation of the bedforms in the flume. (**a**) The numbers show surface water (1), the optode foil (2) and the sediment (3). Photograph of the planar optode and optode imaging unit. (**b**) The numbers show the image unit (4) and the optode foil (5).

The experiments to study the impact of unsteady streamflow on OCRs were carried out in a straight recirculating flume of 260 cm length and 29 cm width inside an air-conditioned laboratory (Fig. 5). The flume was packed with a mixture of natural silica sand and sediment from the Yarqon River, Israel, in the ratio of 3:1^45^. Sediment characteristics are shown in Supplementary Information Table S1. The flume was filled with 400 L of deionized water and flushed 3 times to remove the majority of suspended matter. The water depth in the flume was maintained at an average of 7 cm and at a temperature of $$2{5}^{\circ }$$C $$\pm \ {1}^{\circ }$$C via a water chiller attached to the recirculating system. The pH of the flume water remained between 8 and 8.2 during the experiments. Bedforms were manually formed with a height of 1.5 cm and a length of 12 cm, the length of the stoss side of was 8 cm and the length of the leeside was 4 cm (Fig. 6). These dimensions were chosen as they are typical of bedforms found in lowland sandy streams^46^. The flume was left to run for 48 hours with a streamflow of 9.9 cm sec$${}^{-1}$$ before experiments began. An automated flow control system was built using the Campbell Scientific datalogger (CR1000) connected to an Encom EDS800 power inverter, which controlled the speed of the centrifugal pump driving the water through the flume. The CR1000 was also connected to a Sitrans F M Magflo electromagnic flowmeter which was used to adjust the speed of the pump based on discharge measurements as part of a Proportional-Integral-Derivative loop. The control system was programmed to modulate discharge following a sinusoidal function with a period of 10 hours and an amplitude of $$\pm $$70 L min$${}^{-1}$$ about an average discharge of 120 L min$${}^{-1}$$. The aforementioned discharges produced a streamflow of 9.9 cm sec$${}^{-1}$$$$\pm $$5.8 cm sec$${}^{-1}$$. When changing streamflow between steady and unsteady streamflow a complete 10-hour cycle was run before measurements so that the antecedent streamflow conditions in the flume were the same as the test streamflow conditions. To ensure that experiments were not affected by changing environmental conditions, a reference condition were established and imaged using planar optodes. This reference condition was run periodically throughout the experiment to check that flume environmental conditions were constant and all experiments were conducted within a 2 week period. Vertical fluxes were controlled using a subsurface network of pipes connected to a peristalic pump. Losing and gaining fluxes were balanced with water from a storage tank to ensure that there was no net change in the total volume of water caused by the introduction of a vertical flux. The temperature of the water used to impose losing and gaining fluxes kept at ambient room temperature of $$2{1}^{\circ }$$C. Water temperatures were measured using WTW 3420 Multimeters along with the FDO 925 oxygen sensor. During a preliminary experiment a gaining flux of 10 cm day$${}^{-1}$$ was applied continuously for 48 hours while optode foils were used to confirm that all oxygen in the groundwater was consumed before reaching the bottom of the optode foil which was located 4.5 cm above the bottom of the flume (Fig. 6). Further details on the calibration of the vertical flux system appear in Fox *et al*.^22^.

### HEF measurements

HEF was measured by introducing NaCl as a nonreactive tracer into the surface water and measuring the reduction of electrical conductivity (EC) over time. EC was measured using the LR 925/01 probe and WTW Multi Logger 3430. HEF was calculated based on mass balance equations, which were applied to the initial exchange of salts with the streambed following the method described in Fox *et al*.^22^. The experiments were conducted under steady streamflow and across the range of streamflows that cover the range of unsteady streamflow conditions. We used Eq. 1 to calculate the best fit, and used it to calculate exchange flux under unsteady flow conditions.

1 $${\rm{HEF}}=a{\rm{EXP}}(bx)$$

Where HEF is in cm day${}^{-1}$, $a$ and $b$ are fitting parameters which provided the best results for predicting HEF (cm day${}^{-1}$) at a range of streamflows, *x*, (cm sec${}^{-1}$).

### Optodes

A single planar optode oxygen sensor foil of 14.1 cm in length and 10.5 cm in width was used to measure subsurface oxygen saturation (Presens RPSu4). Detailed design specifications of the optode foils are described in Tschiersch *et al*.^47^. The optodes were installed 24 hours before packing the flume with sediment to allow sufficient time for the adhesive to cure. A two-point calibration was performed at 0 and 100% oxygen saturation in a calibration chamber attached to the flume using a piece of the same planar optode foil. The Presens DU01 Dectector was used with its largest field of view, 40 mm by 32 mm, to acquire images of oxygen concentrations through the glass wall of the flume (Fig. 6). A succession of 15–17 partially overlapping images were captured in less than two minutes. Throughout the duration of the experiment the flume remained covered with a dark curtain to prevent optode bleaching. Oxygen concentration measurements in the stream were taken with a WTW Multi Logger 3430 and an FDO 925 oxygen sensor to confirm that the water in the channel was fully saturated with oxygen and to test the optode calibration.

### Image processing

Optode images were joined to produce a single image of 2991 by 1984 pixels with each pixel representing an area of $4\times 1{0}^{-2}$ cm${}^{2}$. The open source software Hugin version 2015 was used for image stitching (Szeliski 2006). Image stitching consisted of two steps, image registration where overlapping portions of images were identified and image alignment where images were translated or rotated to align overlapping areas. Portions of the image inside of the area of interest where no measurement took place, were assigned the median value of surrounding measurements. The computer vision library OpenCV2 was used to extract pixels above a threshold of 15% oxygen saturation, which was used to define the oxic zone during analysis (see Supplementary Information Fig. S3).

### Data analysis

Subsurface oxygen concentrations were used to obtain the oxygenated area of the bedform (i.e. areas where oxygen saturation was >15%) in the flume experiment. It was assumed that the oxygen distribution was homogeneous along the width of the flume and that the test bedform was representative for all other bedforms in the flume. Thus, to calculate the total V${}_{{\rm{oxic}}}$ in the flume, the area of the oxic zone which was measured using the optode foil was multiplied by the width of the flume and then multiplied by the number of bedforms present in the flume. Oxygen saturation measurements from streamwater were converted to oxygen concentrations based on water temperature and salinity. The oxygen flux into the streambed (O${}_{2}$ mg L per unit streambed) was calculated by multiplying the water flux (cm day${}^{-1}$) into the streambed by the concentration of oxygen in the streamwater. Water flux into the streambed under neutral and gaining conditions is equal to the HEF, while under losing conditions it is the sum of HEF and the losing flux applied. The flux of oxygen to the sediment was divided by the total V${}_{{\rm{oxic}}}$ imaged by the optodes to calculate the OCR in units of mg ${{\rm{O}}}_{2}$ L of oxygenated sediment hr${}^{-1}$. The results of V${}_{{\rm{oxic}}}$ and OCR under steady and unsteady streamflow were compared using the Welch Two Sample t-test due to the unequal number of samples.

2 $$y(t)=A\sin (2\pi ft+\varphi )$$

In order to calculate the delay in response of V${}_{{\rm{oxic}}}$ the sine function, Eq. 2, was fitted to oxygen measurements. Where *y* is the response of V${}_{{\rm{oxic}}}$ at time *T*, *A* is the amplitude of the sine wave, *f* is the frequency and $\varphi$ is the phase shift. The phase shift of the response of V${}_{{\rm{oxic}}}$ relative to the streamflow sinousoid was used to calculate the delay (where negative $\varphi$ indicates a delay).

3 $$NSE=1-\frac{{\sum }_{t=1}^{T}{({Q}_{m}^{t}-{Q}_{o}^{t})}^{2}}{{\sum }_{t=1}^{T}{({Q}_{o}^{t}-\bar{{Q}_{o}})}^{2}}$$

Finally the Nash-Sutcliffe model efficient coefficient (NSE)^48^ was calculated of each set of V${}_{{\rm{oxic}}}$ measurements, Eq. 3 where NSE is the Nash-Sutcliffe model efficiency coefficient, *T* is time, ${Q}_{m}$ are modelled values and ${Q}_{o}$ are observed values. The NSE used to verify that a sine function was appropriate to fit (NSE $\ge$ 85%) the V${}_{{\rm{oxic}}}$ data (Supplementary Fig. S2).

## Supplementary information

Supplementary Information

## Data Availability

The datasets generated during and analysed during the current study are available from the corresponding author on request.
