# Supplementary material for: The effect of unsteady streamflow and stream-groundwater interactions on oxygen consumption in a sandy streambed
Source: Sci Rep. 2019 Dec 24;9:19735. doi: 10.1038/s41598-019-56289-y (PMC6930257; doi:10.1038/s41598-019-56289-y)
Supplement: Supplementary file 1 — Supplementary Information [file 41598_2019_56289_MOESM1_ESM.pdf]

The effect of unsteady streamflow and stream-groundwater interactions on oxygen consumption in a sandy streambed

Jason Galloway[1,2,*], Aryeh Fox[3], Jörg Lewandowski[1,2], and Shai Arnon[3]

[1]Leibniz-Institute of Freshwater Ecology and Inland Fisheries, Department Ecohydrology,Berlin, D-12587, Germany
[2]Humboldt University Berlin, Geography Department, Berlin, D-12489, Germany
[3]Ben-Gurion University of the Negev, The Jacob Blaustein Institute for Desert Research, Zuckerberg Institute for Water Research, Sde-Boker, 84990, Israel
[*]galloway@igb-berlin.de

Additional information

Competing Interests: The authors declare no competing interests.

n	Sediment parameter	Mean	Standard Deviation
n=5	Porosity [%]	0.31	0.01
-	Average mean diameter [µm]	205	-
n=3	Hydraulic conductivity [cm/s]	0.0325	0.00029
n=5	Organic Matter Content (%)	0.31	0.07

Supplementary Information Table S 1: Physical sediment characteristics.

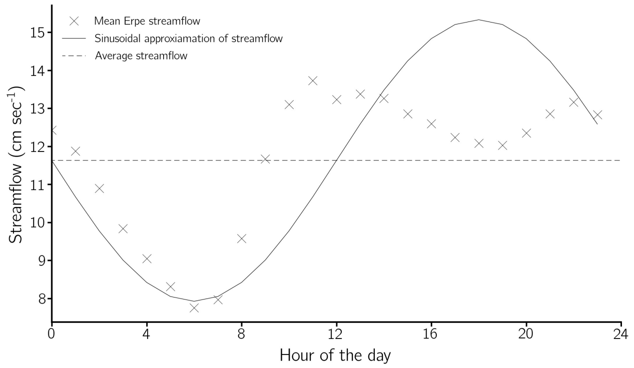

Supplementary Information Figure S 1: Daily average streamflow observed in the river Erpe (crosses), the sinusoidal function used to modulate flume streamflow (solid) and mean streamflow (dashed). Observations took place over a period of 51 days.

q_{gw}	Coefficient a	Exponent b
0	0.84	0.14
-3	0.13	0.23
-6	0	0.43

Supplementary Information Table S 2: Fitted coefficients and exponents for salt tracer hyporheic exchange tests in the presence of various losing fluxes (q_{gw}).

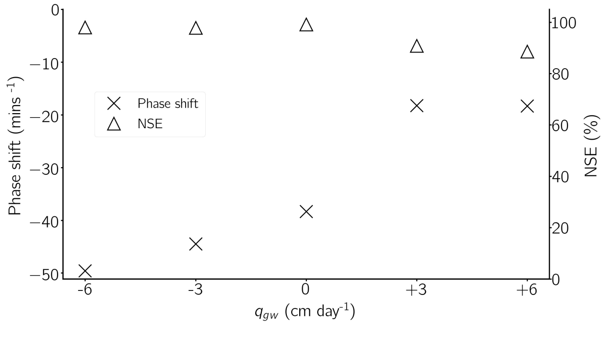

Supplementary Information Figure S 2: Phase shift and Nash-Sutcliffe model efficiency coefficient (NSE) of sine curves fitted to the volume of oxygenated sediment present in the flume in presence of varying groundwater fluxes (q_{gw}). A negative phase shift represents a delay in time.

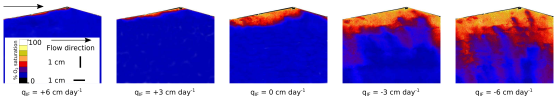

Supplementary Information Figure S 3: Optode image sequence showing subsurface O_2 saturation during steady streamflow and in the presence of various vertical fluxes, q_{gw}.

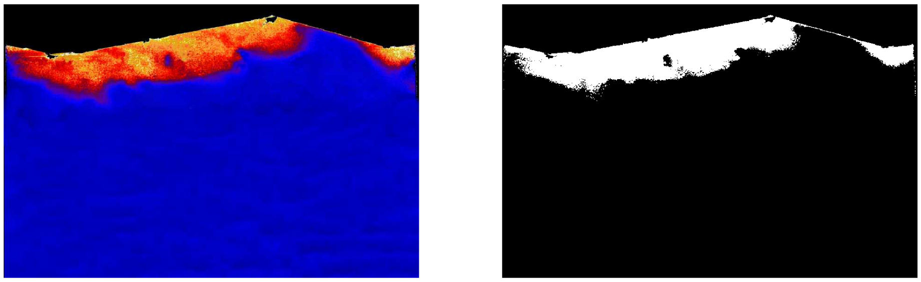

Supplementary Information Figure S 4: Example of optode image (left) and masked image to extract the volume of the oxygenated zone (right).
